# The Use of Radiomic Features to Predict Human Papillomavirus (HPV) Status in Head and Neck Tumors: A Review

**DOI:** 10.7759/cureus.44476

**Published:** 2023-08-31

**Authors:** Judah Glogauer, Avraham Kohanzadeh, Avery Feit, Jeffrey E Fournier, Avraham Zians, Dafna Z Somogyi

**Affiliations:** 1 Department of Pathology and Molecular Medicine, McMaster University, Waterloo, CAN; 2 Medical School, Albert Einstein College of Medicine, Bronx, USA; 3 Department of Pathology and Molecular Medicine, McMaster University, Hamilton, CAN; 4 Department of Diagnostic and Interventional Radiology, Montefiore Medical Center, Wakefield Campus, Bronx, USA; 5 Department of Internal Medicine, Westchester Medical Center, Valhalla, USA

**Keywords:** otolaryngology, cancer of the head and neck, human papillomavirus (hpv), radiology, radiomics

## Abstract

Head and neck cancers represent a significant source of morbidity and mortality across the world. The individual genetic makeup of each tumor can help to determine the course of treatment and can help clinicians predict prognosis. Non-invasive tools to determine the genetic status of these tumors, particularly p16 (human papillomavirus (HPV)) status could prove extremely valuable to treating clinicians and surgeons. The field of radiomics is a burgeoning area of radiology practice that aims to provide quantitative biomarkers that can be derived from radiological images and could prove useful in determining p16 status non-invasively. In this review, we summarize the current evidence for the use of radiomics to determine the HPV status of head and neck tumors.

## Introduction and background

Head and neck cancers constitute a heterogeneous group of malignancies that have a combined worldwide incidence of approximately 600,000 per year and thus represent the sixth most common form of cancer [1,2]. Moreover, head and neck cancers have a poor prognosis compared to other malignancies, with approximately only 40-50% of diagnosed patients surviving after five years [3]. Head and neck cancer sub-types consist of malignancies of the oral cavity, oropharynx, larynx, and hypopharynx, with each sub-type having its own separate group of risk factors and prognostic implications guiding treatment decisions and survival rates [4]. 

One of the most significant risk factors for head and neck cancers, in general, is infection with a high-risk subtype of human papillomavirus (HPV) including types 16, 18, 31, and 33.  The incidence of infection by such viruses has been increasing in certain areas of the Western world, and not coincidentally, the incidence of HPV-associated oropharyngeal cancer has also been increasing over the past decade [5]. This unique risk factor is also notable for prognostic and treatment-related decision-making. 

This review will specifically focus on the determination of HPV positivity as a marker for head and neck malignancies, particularly in terms of staging and management. Other subtypes of head and neck cancer have their own unique risk factors and molecular markers, such as Ebstein Barr virus-positive in nasopharyngeal carcinoma [6], which are beyond the scope of this review. 

HPV-positive head and neck tumors have a better prognosis when compared to HPV-negative tumors [7]. HPV-positive tumors are also managed differently, with HPV-positive tumors showing a better response to both chemotherapy and radiotherapy [8]. While patients with HPV-positive tumors are ultimately treated similarly, there are several differences in the management pathways, including the treatment of T1N1M0 HPV-positive oropharyngeal tumors with surgery or radiation alone. These differences in clinical management underscore the importance of determining the HPV mutation status of the tumor. 

Due to the litany of head and neck cancers with their own distinct risk factors, molecular characteristics, and prognoses, treatment and diagnoses of such malignancies must be tailored to the individual disease, as well as the characteristics of the patients themselves. However, what has become evident over decades of clinical practice and research is the need for better tools to obtain and analyze tumor-related data that could impact management decisions. In some cases, obtaining the necessary data to guide treatment decisions is not feasible for the patient, due to the location of the tumor, or the current health status of the patient. Often what is key to guiding such decisions is the obtaining of a tissue sample to obtain genetic and histologic information about the tumor itself, an area that the budding field of radio-genomics aims to address. In other cases, information about the potential for local recurrence or distant metastasis is not available. It is these specific knowledge gaps, among others, that the field of radiomics aims to address. This review will discuss the field of radiomics, specifically the subfield of radio-genomics, and the potential to use radiomic and radio-genomic analysis to determine the HPV positivity of head and neck tumors. 

## Review

Radiomics: the relevant basics 

A focused and narrative literature review was undertaken by sourcing articles from PubMed and Google Scholar, employing keywords such as "p16 head and neck cancer" and "radiomics." The search was refined by scrutinizing article abstracts, considering the relevance of each study to the central theme of this review: the potential of radiomics in detecting p16 status in head and neck squamous cell carcinoma. Studies utilizing tissue biopsy for p16 status identification were excluded from the scope of this review.

Clinical oncology has long utilized medical imaging for more than just the detection of malignancies. Computed tomography (CT), magnetic resonance imaging (MRI), and positron emission tomography (PET) scans have become key tools for personalized cancer care including: (i) the staging of the tumor to guide management decisions [9], (ii) radiation treatment planning to guide delivery of radioactive material to the diseased area, while sparing normal tissues damaging doses of radiation [10], and (iii) the monitoring of tumor progression to determine future potential risk and treatment decision making [11]. Innovation in the field of medical imaging has thus led to numerous changes in clinical practice, contributing in key ways to patient survival worldwide. These innovations include newer and more advanced hardware for better detection of disease including four-dimensional CT scanners [12], dual-energy CT scanners [13], combined PET/CT [14], novel imaging agents for the higher resolution locating of the diseased areas [15], and novel radiotherapy devices including MRI guided linear accelerators [16]. A further area of innovation in the field of medical imaging is the development of novel methods of image analysis and data acquisition. The field of radiology traditionally involved the acquisition of an image through various modalities, including standard X-ray, CT scan, or MRI, and the determination of clinically relevant data by the trained eye of the radiologist, a physician trained in the clinical aspects of radiological diagnosis [17]. This approach to medical imaging has been termed a “qualitative approach” to image analysis. Another view, one that has become increasingly validated through novel research, is one of “quantitative image analysis”, a view that images provide more than “just pictures”, but that within the image is mineable data that can also be used for clinical decision making. It is this view that underlies the field of radiomics [18]. Within images produced through various modalities, including CT, MRI, and PET scans, there exist numerous types of radiologic features that can be extracted algorithmically and validated statistically to correlate with and/or reflect underlying tumor biology [19], predict the risk of local recurrence [20], predict metastatic risk [21], as well as prognostic factors [22]. The major advantage of radiomics compared to standard radiological analysis is that through high-throughput analysis by automated methods, significant information can be obtained that could not be acquired through human analysis alone. Over the past decade, a growing body of literature has indicated that radiomic features can indeed provide information related to critical clinical factors that standard radiological practice alone can’t match.  

The radiomic approach begins with the acquisition of high-quality images through any number of imaging modalities, including CT, MRI, and PET scans [18]. Next, the region of interest (ROI) is manually, or through an automated program, segmented to create a well-defined area to be studied. In the case of malignancy, this ROI is usually the macroscopic tumor area. Once this region has been segmented, quantitative features are then extracted, which can be used to determine key features of the tumor, that enable clinicians to grade, stage, determine prognosis, and make tumor-specific treatment decisions.  

The quantitative features that have been studied in the context of radiomic extraction can be broken up into two broad groups: (i) semantic and (ii) agnostic features [18]. Semantic features are similar to the details that a radiologist will describe when reporting on an image, features such as “spiculated”, “ground glass”, and “vascular”. Agnostic features are most commonly referred to as “radiomic features”, consisting of quantitative qualifiers, and can be grouped under several categories [23]. The first category is that of first-order statistics, which refers to the distribution of the specific values of three-dimensional areas of the image called voxels. First-order statistics include values such as the mean of the distribution of voxels, the mode, the median, kurtosis, maximum value, and skewness of the distribution. First-order statistics are unrelated to the spatial relationships between the voxels and are entirely focused on the values themselves. Second-order statistics, the second category of agnostic features, refer to so-called “textural” aspects of the ROI, or interrelationships between voxels with similar values. These second-order statistics have proven to be statistically valid measures of intra-tumor heterogeneity, a concept proving to be critical to cancer management in the modern era. The final category of agnostic features in radiomics is that of so-called “higher order” statistics. These features filter through images to determine complicated patterns of voxels containing similar values. Examples of these features include fractal patterns, Minkowski analyses, and wavelets, among others. Numerous studies are being published validating novel radiomic features to various outcomes in multiple types of malignancies [24,25]. This increasing body of radiomic features is being shown to have the potential to help determine prognosis, metastasis, risk of local and distant recurrence, as well as genomic information for many different cancers. 

Often this feature extraction process yields a high number of features, many of which prove to be redundant. It is at this point that researchers will perform several statistical measures to determine which features will prove to be statistically relevant to predict the outcome of interest. Once these statistical procedures have been performed, the statistically relevant features are then compiled into a model that can be used to discriminate between the two groups of interest. Through this process, researchers can construct predictive models that can discriminate between groups regarding a clinically relevant question, including important clinical and molecular information, such as the specific mutational status of a tumor. In the context of head and neck cancer, the HPV mutational status of the tumor can provide significant information to the treating clinician in regard to decision making. Previously, this information could only be provided through immuno-histochemical staining of biopsy tissue, or other molecular methods that required a sample of the tumor such as Next Generation Sequencing or quantitative polymerase chain reaction (qPCR). A number of publications have examined the possibility of utilizing a radiomic approach to determine this information from radiological images. This next section will examine a select group of publications that have focused on this concept. 

HPV, radiomics in head and neck cancer

Many sub-types of head and neck cancer have their own molecular signature, in addition to common mutations seen amongst all head and neck malignancies. The presence or absence of these mutations can be key to determining prognosis and management. General mutations featured by all head and neck cancers include mutations in tumor suppressor genes such as p53, Rb, and APC, proto-oncogenes such as Myc, oncogenes such as Ras, and many more [26]. Mutations in these genes are not specific to head and neck cancers and are also found to be mutated in various other malignancies, including colorectal cancer, lung cancer, and breast cancer among others. In terms of specific genes, for example, oropharyngeal cancers have been linked with mutations in genes related to Notch signaling, such as Notch1 as well as genes related to DNA repair and the ATM signaling pathway [26]. Other head and neck malignancies such as nasopharyngeal cancer have been linked with mutations in specific Ras-related signaling genes such as RASSF1, as well as specific oncogenes such as CCND1 [6]. 

Determining which clinically relevant mutations are present within the tumor is a key aspect of treatment, and often requires invasive tissue sampling. However, as mentioned previously, a significant theme in modern oncology focuses on the limitations of biopsy-based assays. In the event of a patient being able to have a potentially malignant tumor biopsied, the information determined by the pathologist by examining the tissue under the microscope, while potentially useful, cannot take into account the genetic or morphological information of the entire tumor. The need to obtain this information has necessitated the development of other strategies to determine the genetic information of the entire tumor in a noninvasive fashion. The question that remains: Can a radiomic approach determine clinically meaningful genetic information? Head and neck cancer is a key area of malignancy where such an approach could be extremely useful. As previously mentioned, infection with HPV and the subsequent development of a head and neck tumor can lead to a specific subtype of malignancy, with the association of the infecting virus having drastic implications for therapy and prognosis [5]. HPV is notable for inducing the transcription and translation of a novel oncogenic protein p16 which has been demonstrated in both in-vivo and in-vitro models to act on the p53-Rb pathway related to cell cycle progression [5]. Dysregulation of this pathway has been linked with the incidence of other forms of cancers beyond just oropharyngeal cancer. The key players in this pathway, p53 and Rb, when inactivated or degraded, allow for unchecked cellular mitosis, leading to the formation of potentially malignant masses. Indeed, p53 mutation variants have been shown to have prognostic implications for a variety of cancers, including, but not limited to, lung adenocarcinoma, hepatocellular carcinoma, and acute myeloid leukemia. p16, produced from the RNA transcripts derived from HPV DNA, has been shown to degrade p53. In addition, E7, another protein derived from HPV has been indicated to bind to Rb and prevent its functioning. The oncogenic potential of this virus has been shown to be quite significant [5]. 

In terms of prognosis, a p16-positive tumor has a different staging system when compared to a p16-negative tumor. Patients with HPV-positive disease thus have a better prognosis, a better response to specific therapies, and essentially represent a completely different clinical disease compared to HPV-negative disease [27]. Treatment pathways for patients with HPV-associated oropharyngeal cancer are thus quite distinct, with HPV-associated oropharyngeal cancers being shown to be statistically far more likely to respond to radiation therapy as well as chemotherapy compared to non-HPV-associated oropharyngeal cancer. Indeed, current evidence indicates an 82% response rate of HPV-associated malignancies of the oropharynx compared to non-HPV-associated oropharyngeal cancer [28]. Overall survival rates have also been shown to be statistically better in HPV-associated cancers, with overall survival of HPV-related malignancies after two years being 95% compared to 62% for non-HPV-associated oropharyngeal cancer [28]. 

As such, being able to determine the p16 status of a tumor non-invasively proves immensely valuable. In a 2015 study, Buch et al. set out to determine whether a combination of histogram-based values and second-order statistics could help separate p16 positive and negative tumors on CT scans [29]. A retrospective chart review of 40 patients, 29 HPV-positive and 11 HPV-negative, was performed. The primary lesion as the region of interest was contoured by an experienced radiologist and feature extraction was performed, with a number of histogram-based and second-order statistics extracted. Interestingly, when a comparison between the HPV-positive and HPV-negative cohorts was performed, the only feature that was found to be statistically significant in terms of differentiating the two was a histogram-based feature, the median of the intensity histogram values. While initially, several other features were found to be statistically different between the two groups, after performing a false discovery rate (FDR), the researchers only found that the single histogram-based statistic was found to be a potential differentiator between HPV positive and negative lesions. The researchers thus hypothesized that there is a certain degree of uniformity in terms of the delineation of line-based and brightness-based features between HPV-positive and HPV-negative tumors. They also note that the lack of ability to utilize Grey Level Co-Occurrence Matrix (GLCM) type features to differentiate between HPV positive and negative tumors may be due to the lack of detail inherent within CT scans. However, the findings of a different group of researchers conflict with this interpretation. Fujita et al. examined the CT scans of 46 patients with oropharyngeal cancer, 10 of whom had HPV-positive disease [30]. Upon analysis, 10 textural features were found to be statistically different between HPV-positive and HPV-negative oral cavity tumors, and 24 textural features were found to differentiate between HPV positive and negative laryngeal tumors. Of these differentiating features, a number of them (eight in the oral cavity group and 23 in the laryngeal group) were of the second-order statistical nature, with GLCM-based features in particular showing differentiating power in both groups. While this study had no histopathological correlation and a small sample size, it indicated the possibility that second-order statistics could prove useful in differentiating HPV-positive and HPV-negative disease. 

Further publications indicate the utility of radiomics-based CT analysis in clinical practice in the ability to determine the p16 status of oropharyngeal cancer. Remarkably, some of these models show significantly better predictive value when compared to radiologists. Ranjbar et al. developed a texture-analysis-based model from CT scans of oropharyngeal cancer patients and compared this model to classification attempts at HPV status by two trained neuroradiologists [31]. A total of 107 patient CT scans were reviewed by both radiologists who attempted to classify the p16 status of each patient based purely on the CT scan. However, it was recognized that the ability to determine HPV status purely from the appearance of the tumor itself without referencing margins, nodal status, and metastatic status is quite difficult for even a trained neuro-radiologist. After the classification had been attempted by the neuro-radiologists, texture analysis was performed, and a predictive model was developed. This model utilized primarily second-order statistics and when compared to the classification attempts by the radiologists, was found to be 75.7% accurate, higher than either radiologist (44.9% and 55.1% accuracy, respectively). Bogowicz et al. also developed a model to differentiate HPV-positive and HPV-negative tumors utilizing CT scans [20]. Their ultimate model was constructed from four features including first-order statistics such as standard deviation and second-order statistics utilizing grey level analysis. Their model had sufficiently high specificity and sensitivity to predict HPV status fairly accurately, and their signature was found to be predictive independently from a local control radiomic signature that they were developing in the same study. In addition, by analyzing their radiomic signature, they found that tumors that were HPV positive had a more homogenous CT density distribution when compared to HPV-negative tumors. This seems to fit with the hypothesis that tumors that are more homogenous have a better overall prognosis, a phenomenon seen when comparing HPV positive and negative tumors.

Yu et al. found a similar conclusion, having extracted 1,683 features from the CT scans of 315 oropharyngeal cancer patients, and determined a predictive model utilizing two features, mean distribution and spherical disproportion [32]. Mean distribution, which measures the mean width of the tumor, was found to be significantly lower in patients with HPV-positive tumors, and spherical disproportion, which measures how complicated the shape of the tumor is (the ratio of the surface area of the region of interest to the surface area of a sphere of the same size), was also found to be significantly lower in HPV positive tumors. This finding is in keeping with the hypothesis that HPV-positive tumors are generally less complex and more homogenous than HPV-negative tumors, which may account for the more favorable prognosis of HPV-positive disease. A further study by Leijenaar et al. bolstered this conclusion utilizing second-order statistics (gray-level analysis), finding that the gray-level size zone matrix with a small zone emphasis was decreased in HPV-positive tumors compared to HPV-negative tumors, a finding that was interpreted as reflecting increased homogeneity [33].

While significantly more research will need to be performed to adequately conclude that these features reflect differences in homogeneity, these publications reflect a growing consensus that these findings are accurate. Leijenaar et al. also noted significant histopathological differences may be present to differentiate between HPV positive and negative tumors, with HPV-positive tumors displaying more lobular growth, infiltrating lymphocytes, and well-differentiated cells. They note that in order to correlate radiomic features with histopathological ones, a more in-depth study would need to be performed utilizing a surgical cohort. 

MRI radiomics and the determination of HPV status in head and neck cancers 

In addition to the aforementioned studies linking CT radiomic analysis and HPV status, another publication examined the possibility of using MRI-based radiomics to determine p16 status. Ravanelli et al. looked at the diffusion-weighted MRI scans of 59 patients with oropharyngeal cancer with the intent of determining the association between HPV status and various MRI-related textural analyses, as well as apparent diffusion coefficient (ADC) [19]. While before performing a false discovery rate analysis, several textural features appeared to discriminate statistically between HPV positive and negative tumors. After the analysis was run, only ADC was found to be a discriminating feature. A number of hypotheses were proposed as to why ADC was a discriminating feature between HPV positive and negative tumors, but due to the lack of research on the topic, no conclusions could be drawn.

Suh et al. looked at the MRI scans of 60 patients with oropharyngeal squamous cell carcinoma and extracted features to develop models to determine HPV-positive status [34]. They extracted 1618 features from the tumors and pared these using the least absolute shrinkage and selection operator and utilized three machine learning classifiers to determine the area under the curve (AUC) for each classifier in terms of how each model determined HPV status. All three of the models showed AUC of 0.70 or greater, indicating the potential value of machine learning classifiers utilizing radiomic features to determine the HPV status of oropharyngeal tumors. This work was further bolstered by Sohn et al., who utilized MRI data to determine the HPV status of 62 patients with oropharyngeal squamous cell carcinoma [35]. The retrospective data was divided into a training set of 43 and a test set of 19 patients. Ultimately, from 170 extracted features, six radiomic features were found to be strongly associated with HPV positivity and this model ultimately proved to have a high level of accuracy in predicting HPV-positive status in these patients with an area under the curve of 0.982 on the training set and 0.744 on the test set. However, due to the small sample size, the researchers noted the importance of more research before drawing any conclusions. More research on MRI textural and radiomic analysis will be required before any radiomic signature can be validated. 

These studies, when compiled, indicate a clear possibility to utilize radiomics-based analyses to determine the HPV status of head and neck cancers. This approach could prove vital to the clinician when trying to make treatment decisions for patients. Treatment and prognostic decisions can be extremely different depending on the p16 status of the tumor. Such approaches could thus be extremely valuable in the modern-day oncology world, revolutionizing the manner in which diagnoses and treatment decisions are made by clinicians. 

## Conclusions

The prospect of determining the genetic makeup of a tumor through non-invasive imaging-based techniques represents an interesting, yet significantly understudied area of investigation. While significant research has been conducted into this area, there remain large knowledge gaps that will need to be addressed before radiomics-based genetic determination can be integrated into clinical head and neck oncology practice. The clinical relevance of HPV mutations has been discussed, with the possibility to guide future diagnoses and treatments in the world of oncology. While previously these mutations were detected with invasive procedures leading to a biopsy, the prospects of determining this information non-invasively are clearly enticing both from the perspective of the patient and the clinician. Some patients can simply not tolerate a biopsy, while others have tumors that simply cannot be reached with conventional surgical techniques. These strategies can also be extremely effective in terms of patient morbidity, sparing them the discomfort associated with a biopsy. This review discussed the radiomic and radio-genomic approaches associated with determining HPV mutation status in the context of head and neck cancer. Other potential mutations remain within the scope of future research, with the goal of being able to develop radiomic signatures to determine such mutations non-invasively now within grasp. With this being said, the field of radio-genomics appears to be an extremely promising one with a bright future ahead. 
